# Knowledge and use of HIV pre-exposure prophylaxis among men who have sex with men in Berlin – A multicentre, cross-sectional survey

**DOI:** 10.1371/journal.pone.0204067

**Published:** 2018-09-13

**Authors:** Ricardo Niklas Werner, Matthew Gaskins, Jens Ahrens, Heiko Jessen, Frank Kutscha, Regina Mosdzen, Wolfgang Osswald, Dirk Sander, Sven Schellberg, Kai Schwabe, Thomas Wünsche, Corinna Dressler, Mary Sammons, Alexander Nast

**Affiliations:** 1 Charité–Universitätsmedizin Berlin, corporate member of Freie Universität Berlin, Humboldt-Universität zu Berlin and Berlin Institute of Health; Department of Dermatology, Venereology and Allergy; Division of Evidence-Based Medicine (dEBM), Berlin, Germany; 2 Berliner Aids-Hilfe e.V., Berlin, Germany; 3 Praxis Jessen^2^ + Kollegen, SHC–Sexual Health Center Berlin, Berlin, Germany; 4 Schwulenberatung Berlin gGmbH, Berlin, Germany; 5 Fixpunkt Berlin e.V., Berlin, Germany; 6 Mann-O-Meter e.V., Berlin, Germany; 7 Deutsche AIDS-Hilfe e.V., Berlin, Germany; 8 Novopraxis Berlin GbR, Berlin, Germany; 9 Praxis Wünsche Berlin, Berlin, Germany; David Geffen School of Medicine at UCLA, UNITED STATES

## Abstract

**Background:**

HIV pre-exposure prophylaxis (PrEP) has likely contributed to large decreases in HIV incidence among men who have sex with men (MSM) in several major cities. Berlin has seen a smaller decline, and affordable PrEP has been accessible through formal channels in Germany only since autumn 2017. We aimed to investigate knowledge and use of PrEP among MSM in Berlin, and factors predictive of a desire to use PrEP and history of PrEP use.

**Methods:**

Multicentre, paper-based, self-administered survey of adult MSM whose HIV status was negative or unknown at time of participation. Data were collected from 1 October 2017 to 2 April 2018.

**Results:**

473 of 875 questionnaires were returned (response rate 54.1%; mean age 37.4 years, range 18–79). 90.0% of participants were aware of PrEP and, of these, 48.2% felt well informed about it. Among the 17.2% of participants reporting PrEP use, 59.3% indicated obtaining some or all of it from informal sources. 23.7% of those with no history of PrEP use reported having condomless anal intercourse (CAI) with two or more partners over the past six months. Worries about side effects, cost, not having a doctor who prescribes it, and a lack of information were the most frequently reported barriers to PrEP use. A desire to use PrEP and history of PrEP use were associated in our multivariable model with having multiple CAI partners. A history of PrEP use was associated with having a university degree, one or two parents born outside Germany, or friends living with HIV.

**Conclusions:**

We found high awareness of PrEP among MSM in Berlin, but also a strong need for more education on its pros, cons and proper use. The frequency of informal PrEP use was also high, raising urgent individual and public health concerns. Policy makers need to consider recent calls to improve access to PrEP and PrEP education through regular health services.

## Introduction

HIV pre-exposure prophylaxis, or PrEP, is a biomedical form of HIV prevention that has demonstrated high efficacy and safety in clinical trials [1–4] and cohort studies [5–15]. In 2017 the Centers for Disease Control and Prevention (CDC) in the United States issued an updated clinical practice guideline recommending PrEP for men who have sex with men (MSM) and who report having had a bacterial sexually transmitted infection (STI), anal sex without condoms outside a monogamous relationship with an HIV-negative partner, or both within the past six months [16]. The results of a modelling study from 2016 suggest that achieving 40% coverage of indicated MSM would avert 33% of infections expected in the US over the next decade [17]. Indeed, increased use of PrEP is thought to have already contributed to substantial declines in HIV incidence among MSM in London [18], San Francisco [19] and New South Wales, Australia [20].

To become an effective part of HIV prevention strategies, PrEP must be made accessible to the populations at highest risk of HIV infection, such as MSM. However, while awareness of PrEP among MSM is generally increasing [21–25], it varies widely across geographies [26,27], as well as socioeconomic and ethnic groups [28,29]. Likewise, the willingness of MSM to use PrEP is influenced by various factors, including cost, perceived level of protection against HIV infection, adverse effects and socioeconomic status [30–32].

In Germany, the incidence of HIV among MSM has decreased since 2013, falling from 2500 new cases that year to an estimated 2100 in 2016 [33]. This decline has been attributed primarily to the use of HIV treatment as a form of prevention [33]. Around 20% of new cases of HIV among MSM in Germany in 2016 were diagnosed in immigrants, with central Europe, western Europe and South America being the most frequent regions of origin [34]. The German states with the highest HIV incidence were the city-states of Berlin and Hamburg, both of which saw 10.1 new cases of HIV per 100,000 population compared to an incidence of 4.2 per 100,000 in Germany as a whole [34].

Berlin joined the Fast-Track Cities initiative of the Joint United Nations Programme on HIV/AIDS (UNAIDS) in 2016 and, in doing so, committed to attain the 90-90-90 and zero stigma and discrimination targets. In addition to its major goal of rapidly expanding the use of HIV treatment as a highly effective form of prevention [35], the initiative recommends improved and more widespread implementation of other preventive strategies, such as PrEP [36]. The current Berlin state government is planning a model project to deliver free PrEP services to a limited number of people who are not able to afford these themselves [37], satisfying some of the demands of local HIV counselling centres and NGOs [38].

Despite these commitments and plans, very little information is available on what MSM in Germany know about PrEP, the extent to which and how they use it, and the attitudes they have towards it. In particular, there is no information of this nature specifically for Berlin. The aim of our study was therefore to survey MSM attending HIV specialist practices or HIV testing and counselling centres on these topics and to identify barriers, enablers and other factors associated with participants’ desire to use PrEP and any history of PrEP use. Data of this nature from Berlin can provide a useful comparison to the situation in cities such as London or Paris, where the implementation of PrEP is already well underway.

## Materials and methods

### Study design

We conducted a cross-sectional, multicentre survey of MSM attending HIV specialist practices or HIV testing and counselling centres in Berlin using an anonymous, self-administered, paper-based questionnaire. The study protocol was approved by the institutional ethics committee of Charité–Universitätsmedizin Berlin (EA1/162/17, 28 September 2017). Participation was voluntary and all participants gave verbal informed consent in English or German before filling in the questionnaire. We did not provide any incentives to the centres or participants to take part in the study.

### Sampling methods and settings

MSM were eligible to take part in the survey if they were aged 18 years or older and had a self-reported negative or unknown HIV serostatus at the time of participation. Data were collected from 1 October 2017 to 2 April 2018. Because we aimed to recruit a heterogeneous sample of MSM in Berlin, we collected data in various settings: HIV and STI testing and counselling centres for MSM and HIV specialist practices. The former are walk-in centres offering low-threshold, anonymous counselling on legal and health issues, as well as testing for HIV and STIs. They are not permitted to prescribe medication. We invited all of these centres in Berlin (n = 4) to participate in our study. HIV specialist practices in Berlin are owned and staffed by doctors, and visiting them usually requires an appointment. They provide a range of generalist and sexual health care to LGBTI+ people whether or not they are living with HIV. We invited a total of 11 such practices from seven different neighbourhoods across Berlin to participate in our study. These were chosen purposively based on their geographic spread and our knowledge that they had participated in other research related to HIV.

Counsellors invited eligible clients to participate in the survey if they were seeking STI or HIV tests or counselling. Patients at the HIV specialist practices were selected by participating doctors, who had been asked to include every eligible patient consecutively regardless of the reason for the patient consultation. The questionnaire was prefaced with information about PrEP and our survey.

### Content and format of the questionnaire

We designed a two-page questionnaire consisting mostly of closed multiple-choice questions with single or multiple answers allowed. The questions covered the following topics, all of which focused on the perspective of the participating MSM:

- awareness of PrEP and sources of information about it;
- desire to use PrEP and history of PrEP use;
- barriers to PrEP use, including perceived risks;
- preferences for dosage regimen and route of administration;
- anticipated impact of taking PrEP on the participants’ use of condoms; and
- attitudes towards pricing and reimbursement through public insurance.

In addition, we asked questions about participants’ sexual behaviour and HIV risk (date of last HIV test, diagnosis of any STI in the past six months, role in anal sex, number of anal sex partners in the past six months, number of anal sex partners without condoms in the past six months). We also collected sociodemographic data (age, place of residence, education, financial situation and family origins). The last of these variables was chosen to capture information on whether participants had a family or personal history of immigration to Germany.

Additionally, the questionnaire contained an open-ended question focusing on the motivation behind participants’ use of, or desire to use, PrEP. These data will be reported elsewhere. The questionnaire was available in German and English, and the full versions are available as supporting information (S1 and S2 Files).

### Sample size and statistical methods

No formal sample size calculations were performed. Based on considerations of feasibility, we aimed to collect data from 400 to 600 participants. We used descriptive statistics to summarise sample characteristics and Pearson’s chi-squared test to measure the association among pre-selected categorical variables. For the latter analyses, we applied a Bonferroni-adjustment to account for multiple testing (alpha level at 0.005). Additionally, we used multivariable logistic regression to identify predictors of having a desire to use PrEP or a history of PrEP use. Odds ratios and their respective 95% confidence intervals were used to quantify the effects. To select variables for our multivariable model, we compiled the following initial working set of potential predictors in which we had a priori interest based on background knowledge: age, financial situation, education, family origins, sexual risk behavior, self-perceived risk, having peers living with HIV, and perceived barriers and risks of PrEP. For pragmatic reasons of reporting and traceability, we subsequently screened these using simple (i.e., univariable) logistic regression and included in the multivariable model those variables that were associated with the respective dependent variable at a p-value cut-off point of 0.075 following the approach described by Bursac et al. [39]. We later conducted a sensitivity analysis with all variables of a priori interest to ensure that important adjustment variables had not been overlooked. Missing cases were excluded in a listwise fashion.

To avoid collinearity of independent variables related to different measures of sexual risk behaviour in our logistic regression models, we created a new variable comprising four groups as shown in Table 1. In doing so, we aimed to approximate roughly the indications for PrEP use recommended by the CDC for MSM. We chose “two or more partners” rather than “one” as our cut-off point to account for the possibility that participants who reported condomless anal intercourse (CAI) with one partner might be describing CAI within a monogamous partnership. We did not distinguish between receptive or insertive CAI because the CDC indications for PrEP use for MSM do not do so either.

**Table 1:** Definitions of sexual risk behaviour groups, according to self-reported number of condomless anal intercourse partners and diagnosis of any sexually transmitted infection over the past six months.

Label for sexual risk behaviour	Definitions (referring to the past six months)
“Highest risk (CAI + STI)”	Reported having had CAI with two or more partners and a diagnosis of any STI
“Higher risk (CAI)”	Reported having had CAI with two or more partners but no STI diagnosis
“Higher risk (STI)”	Reported having had a diagnosis of any STI but not CAI with two or more partners
“Low risk”	Did not report having had an STI diagnosis or CAI with two or more partners

CAI, condomless anal intercourse; STI, sexually transmitted infection.

IBM SPSS Version 22 was used for the descriptive statistics and cross-tabulations, whereas Stata SE 14.2 (StataCorp) was used to estimate the regression models.

## Results

All of the HIV and STI testing and counselling centres in Berlin (n = 4) chose to participate in the study. Of the 11 HIV specialist practices invited to participate, a total of six elected to take part. The participating centres handed out 875 questionnaires, of which 473 were returned, yielding a response rate of 54.1%. We excluded three participants because they had indicated in the questionnaire that they were living with HIV. This left 470 questionnaires for further analysis.

### Demographic data

Of the 470 questionnaires in our analysis sample, 84.9% were in German. The mean age of the participants was 37.4 years (SD: 11.9; range: 18–79 years), and 94.0% indicated that they lived in Berlin. Around two-thirds (65.3%) of the participants had a university degree, and 87.4% described their financial situation as having “enough money” or “more than enough money” to pay for the things they need. One third of the participants reported either that one or two of their parents (14.9%) or that they themselves (23.8%) had been born outside Germany. One quarter of the participants (24.9%) stated that they had no friends or acquaintances living with HIV, whereas 35.5% and 49.6% reported having acquaintances or friends living with HIV, respectively.

### Sexual risk behaviour

Referring to the past six months, 17.4% of the participants stated that they had been diagnosed with an STI, 68.1% that they had had anal sex with two or more partners, and 32.1% that they had had anal sex with two or more partners without using a condom, respectively. According to our sexual risk behaviour stratification, 58.9% were categorized as “low risk”, 6.4% as “higher risk (STI)”‘, 22.1% as “higher risk (CAI)”, and 11.1% as “highest risk (CAI + STI)”. Seven participants could not be assigned to a category due to missing information for either the number of CAI partners or the diagnosis of an STI in the past six months. Among participants who reported never having used PrEP, almost one quarter (90/379) indicated that they had had CAI with two or more partners in the past six months.

When asked whether the sex they have is always as safe as they would like it to be, 66.0% of all participants agreed or strongly agreed with the statement and 18.9% disagreed or strongly disagreed. Table 2 gives an overview of the demographic and sexual risk behaviour data.

**Table 2:** Demographic data and sexual risk behaviour; total sample and subsamples according to type of centre.

		Total sample	Type of centre	
			Counselling centres^1^	Doctor practices^2^
**N**		470	221	249
**Age**				
	Mean (SD)	37.4 (11.9)	32.9 (8.0)	41.4 (13.2)
	Min; Max	18–79	18–59	19–79
**Highest degree or level of school (N, %)**				
	Primary education	0	0	0
	Secondary education up to year 10*	42 (8.9%)	8 (3.6%)	34 (13.7%)
	Secondary education with apprenticeship	23 (4.9%)	5 (2.3%)	18 (7.2%)
	Secondary education up to year 12**	89 (18.9%)	44 (19.9%)	45 (18.1%)
	University degree	307 (65.3%)	160 (72.4%)	147 (59.0%)
	Not stated	9 (1.9%)	4 (1.8%)	5 (2.0%)
**Financial situation (N, %)**				
	Not always enough money	51 (10.9%)	23 (10.4%)	28 (11.2%)
	Enough money	205 (43.6%)	95 (43.0%)	110 (44.2%)
	More than enough money	206 (43.8%)	99 (44.8%)	107 (43.0%)
	Not stated	8 (1.7%)	4 (1.9%)	4 (1.6%)
**Place of residence (N, %)**				
	Berlin	442 (94.0%)	204 (92.3%)	238 (95.6%)
	Other city in Germany	10 (2.1%)	4 (1.8%)	6 (2.4%)
	Small town / rural area in Germany	4 (0.9%)	3 (1.4%)	1 (0.4%)
	Other country	8 (1.7%)	7 (3.2%)	1 (0.4%)
	Not stated	6 (1.3%)	3 (1.4%)	3 (1.2%)
**Family origins (N, %)**				
	Participants & both parents born in Germany	281 (59.8%)	112 (50.7%)	169 (67.9%)
	One parent born outside Germany	32 (6.8%)	19 (8.6%)	13 (5.2%)
	Both parents born outside Germany	38 (8.1%)	25 (11.3%)	13 (5.2%)
	Participant born outside Germany	112 (23.8%)	62 (28.1%)	50 (20.1%)
	Not stated	7 (1.5%)	3 (1.4%)	4 (1.6%)
**Current HIV status (N, %)**				
	HIV negative	406 (86.4%)	171 (77.4%)	235 (94.4%)
	Not sure	52 (11.1%)	41 (18.6%)	11 (4.4%)
	Not stated	12 (2.6%)	9 (4.1%)	3 (1.2%)
**STI diagnosis in the past six months (N, %)**				
	No	381 (81.1%)	183 (82.8%)	198 (79.5%)
	Yes	82 (17.4%)	34 (15.4%)	48 (19.3%)
	Not stated	7 (1.5%)	4 (1.8%)	3 (1.2%)
**Role when having anal sex (N, %)**				
	No anal sex	21 (4.5%)	2 (0.9%)	19 (7.6%)
	Bottom only	37 (7.9%)	19 (8.6%)	18 (7.2%)
	More bottom than top	91 (19.4%)	48 (21.7%)	43 (17.3%)
	Top and bottom (versatile)	141 (30.0%)	66 (29.9%)	75 (30.1%)
	More top than bottom	99 (21.1%)	47 (21.3%)	52 (20.9%)
	Top only	72 (15.3%)	33 (14.9%)	39 (15.7%)
	Not stated	9 (1.9%)	6 (2.7%)	3 (1.2%)
**Number of anal sex partners in the past six months (N, %)**				
	None	55 (11.7%)	10 (4.5%)	45 (18.1%)
	1	80 (17.0%)	36 (16.3%)	44 (17.7%)
	2 to 5	142 (30.2%)	85 (38.5%)	57 (22.9%)
	6 to 10	79 (16.8%)	38 (17.2%)	41 (16.5%)
	More than 10	99 (21.1%)	45 (20.4%)	54 (21.7%)
	Not stated	15 (3.2%)	7 (3.2%)	8 (3.2%)
**Number of anal sex partners without using condom in the past six months (N, %)**				
	None	174 (37.0%)	68 (30.8%)	106 (42.6%)
	1	134 (28.5%)	79 (35.7%)	55 (22.1%)
	2 to 5	109 (23.2%)	50 (22.6%)	59 (23.7%)
	6 to 10	23 (4.9%)	10 (4.5%)	13 (5.2%)
	More than 10	19 (4.0%)	6 (2.7%)	13 (5.2%)
	Not stated	11 (2.3%)	8 (3.6%)	3 (1.2%)

STI, sexually transmitted infection.

^1^Counselling centres: Fixpunkt e.V., Mann-O-Meter e.V., Berliner AIDS-Hilfe e.V., Pluspunkt / Schwulenberatung Berlin gGmbH (listed in descending order according to number of returned questionnaires).

^2^Practices: Gemeinschaftspraxis Dietmar Schranz und Klaus Fischer, Praxis Jessen^2^ + Kollegen, Praxis Wünsche, Ärztezentrum Nollendorfplatz, Praxiszentrum Kaiserdamm, Novopraxis Berlin GbR (listed in descending order according to number of returned questionnaires).

*or similar.

**for example A levels, high school diploma, German “Abitur”.

### Awareness of PrEP and sources of information

In total, 90% of participants (n = 423) reported already being aware of PrEP. Of these, 48.2% agreed or strongly agreed with the statement that they were well informed about PrEP, whereas 31.9% disagreed or strongly disagreed. Their sources of knowledge about PrEP (multiple answers allowed) were friends or acquaintances (61.7%), magazines, journals or blogs (57.4%), dating apps or platforms (34.0%), doctors (22.7%), counselling centres (13.9%), and others (10.6%). Doctors were named as a source of information about PrEP significantly more often by participants in the “highest risk (CAI + STI)” sexual risk behaviour category than by other participants (42.3% vs. 18.4%, p<0.001). This was not the case with counselling centres, however (17.3% vs. 12.1%, p = 0.291).

### Barriers to PrEP use

Two-thirds (65.6%) of the survey participants agreed or strongly agreed with the statement that PrEP is a safe way to prevent infection with HIV. Agreement was significantly more common among participants who had indicated that they were well informed about PrEP (p<0.001). Participants attributed the following risks to the use of PrEP (multiple answers allowed): A higher risk of getting infected with other STIs (64.3%), mild or temporary side effects (43.6%), severe or permanent side effects (19.8%), a higher risk of getting infected with HIV (6.2%), and other risks (5.1%). After we applied a Bonferroni-adjusted alpha-level (p<0.005) to account for multiple comparisons across survey items, however, the only differences between the well-informed versus not- well-informed groups that remained significant were those for the items “Higher risk of getting infected with other STIs” and “Not sure” (Table 3).

**Table 3:** Participants’ perception of risks of PrEP use, by self-reported level of knowledge about PrEP.

What risks do you see for people who use PrEP? (multiple answers allowed)			
	*“I am well informed about PrEP”*		p value^§^
	Agree or strongly agree (N = 210)	Disagree or strongly disagree (N = 166)	
None	10 (4.8%)	2 (1.2%)	0.051
Mild / temporary side effects	106 (50.5%)	64 (38.6%)	0.021
Severe / permanent side effects	40 (19.0%)	36 (21.7%)	0.527
Higher risk of getting infected with HIV	8 (3.8%)	14 (8.4%)	0.058
Higher risk of getting infected with other STIs	156 (74.3%)	96 (57.8%)	0.001
Other risks	14 (6.7%)	4 (2.4%)	0.055
Not sure	7 (3.3%)	41 (24.7%)	< .001

STI, sexually transmitted infection.

^§^From Chi-squared tests of the null hypothesis that there is a no significant difference between the expected frequencies and the observed frequencies in the categories.

Among participants without a history of PrEP intake (n = 387), the following were named as circumstances under which they would consider using PrEP (multiple answers allowed): if they had fewer worries about side effects (47.3%), if it were cheaper (39.8%), if a doctor prescribed it (31.8%), if they had more information (31.3%), and other circumstances (3.7%).

### Desire to use PrEP

**Fig 1:**
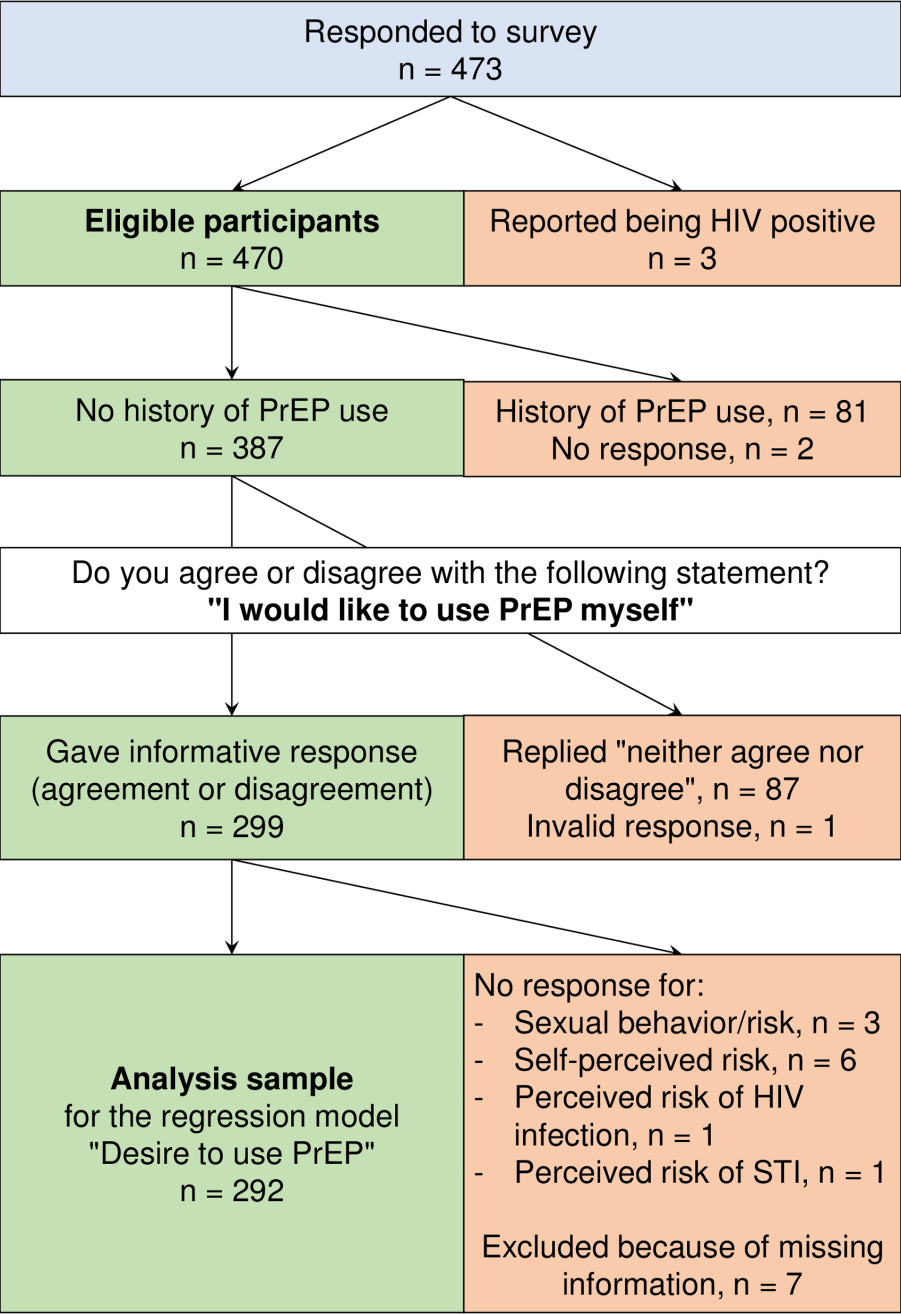
Response tree for the multivariable regression model of desire to use PrEP.

Among participants with no history of PrEP use (n = 387), 42.4% agreed or strongly agreed with the statement that they would like to use PrEP themselves, whereas 34.8% disagreed or strongly disagreed. In our univariable logistic regression models, the following variables were significantly positively associated with the desire to take PrEP: belonging to the “higher risk (CAI)” or “highest risk (CAI + STI)” categories for sexual risk behaviour; perceived riskiness of own sexual behaviour; and expressing the need to have a doctor who prescribed PrEP. Attributing to PrEP a higher risk of getting infected with STIs was significantly negatively associated with the desire to take PrEP. In our multivariable model, the following factors were significant positive predictors of the desire to take PrEP: belonging to the “higher risk (CAI)” or the “highest risk (CAI + STI)” category; and having expressed the need to have a doctor who prescribed PrEP. The one significant negative predictor was having attributed to PrEP a higher risk of getting infected with other STIs (Table 4). A response tree for the multivariable regression model for the desire to use PrEP is shown in Fig 1.

**Table 4:** ORs and 95% CIs for expressing a desire to use PrEP, by sexual risk behaviour, perceived riskiness of own sexual behaviour, and barriers and risks attributed to PrEP intake.

			Participants expressing a desire to use PrEP		Crude OR	Adjusted OR^†^
Participant characteristics		*N*^‡^	*n* (%)	*p* value^§^	(95% CI)	(95% CI)
**Sexual risk behaviour (past six months)**				<0.001		
	No STI; no multiple* CAI partners	193	86 (44.6%)		Reference	Reference
	STI; no multiple* CAI partners	17	7 (41.2%)		0.85 (0.31–2.33)	1.02 (0.34–3.05)
	No STI; multiple* CAI partners	66	52 (78.8%)		4.58 (2.33–9.00)	3.77 (1.84–7.69)
	STI; multiple* CAI partners	20	19 (95.0%)		23.07 (3.03–175.93)	17.22 (2.18–136.14)
**Perceived riskiness of own sexual behaviour: “When I have sex, it is always as safe as I’d like it to be”**				<0.001		
	Strongly disagree	9	6 (66.7%)		Reference	Reference
	Disagree	51	39 (76.5%)		1.27 (0.22–7.39)	2.16 (0.4–11.64)
	Neither agree nor disagree	37	27 (73.0%)		1.16 (0.19–7.04)	2.63 (0.46–14.94)
	Agree	123	64 (52.0%)		0.44 (0.08–2.37)	1.31 (0.26–6.44)
	Strongly agree	73	27 (37.0%)		0.23 (0.04–1.28)	0.77 (0.15–3.90)
**“If a doctor prescribed it”**				0.012		
	Not selected as a circumstance under which participant would use PrEP	202	99 (49.0%)		Reference	Reference
	Selected as a circumstance under which participant would use PrEP	97	65 (67.0%)		1.96 (1.17–3.28)	2.44 (1.36–4.37)
**“A higher risk of getting infected with HIV”**				0.078		
	Not selected as risk seen for people using PrEP	282	158 (56.0%)		Reference	Reference
	Selected as risk seen for people using PrEP	16	5 (31.3%)		0.38 (0.13–1.14)	0.34 (0.10–1.11)
**“A higher risk of getting infected with other STIs”**				0.053		
	Not selected as risk seen for people using PrEP	120	76 (63.3%)		Reference	Reference
	Selected as risk seen for people using PrEP	178	87 (48.9%)		0.53 (0.32–0.87)	0.54 (0.31–0.92)

CAI, condomless anal intercourse; CI, confidence interval; OR, odds ratio; PrEP, HIV pre-exposure prophylaxis; STI, sexually transmitted infection. P-values from joint Wald tests of the null hypothesis that there is no variation across a category for the univariate and multivariate regression models were <0.0001 and 0.0002 for sexual risk behaviour, <0.0001 and 0.0576 for perceived riskiness of own sexual behaviour, 0.0095 and 0.0028 for doctor prescription as a pre-condition for PrEP use, 0.074 and 0.0748 for attributing to PrEP a higher risk of getting infected with HIV, and 0.0105 and 0.0243 for attributing to PrEP a higher risk of getting infected with other STIs, respectively.

^†^Multivariable analysis adjusting for sexual risk behaviour, perceived riskiness of own sexual behaviour, having a doctor who prescribes PrEP, and risk of HIV and STI attributed to PrEP intake.

^‡^The sample excludes patients who were missing information on the relevant variables. Fig 1 gives an overview of participants included and excluded in the regression model.

^§^From Chi-squared tests of the null hypothesis that there is a no significant difference between the expected frequencies and the observed frequencies in one or more categories (e.g., across sexual risk behaviour groups).

*"multiple" was defined as reporting having had two or more CAI partners in the past six months.

### History of PrEP use and sources of PrEP

The majority of participants (82.3%) had never used PrEP themselves. Of the 81 (17.2%) who had, 46.9% reported using it continuously, 13.6% using it on-demand and 39.5% using or having used it but not on a regular basis. Asked about the source of their PrEP (multiple answers allowed), 44.4% of the 81 participants reported obtaining a prescription from their doctor, 35.8% importing it from another country, 18.5% using pills originally prescribed for post-exposure prophylaxis (PEP), 11.1% using pills from a friend’s HIV medication, and 4.9% using other ways to obtain the medication. Only 32.1% of participants who had a history of PrEP intake reported using a private prescription as their only source of PrEP, and 59.3% reported that they had obtained some or all of their PrEP by means other than a private prescription. This latter number rises to 64.8% if we exclude those who did not answer this question (n = 7).

**Fig 2:**
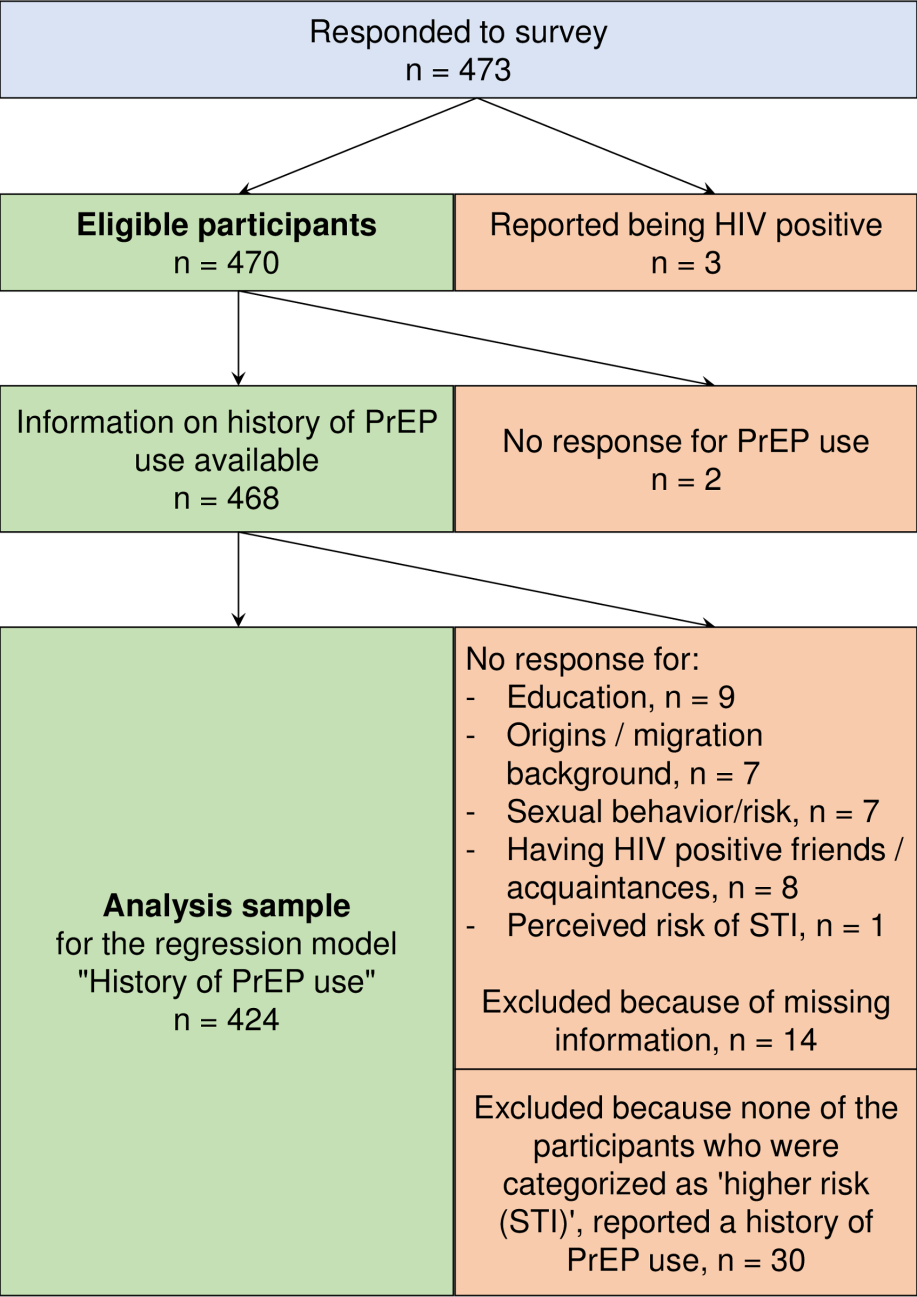
Response tree for the multivariable regression model for history of PrEP use.

In our univariable logistic regression models, the following variables were significantly positively associated with a history of PrEP use: having a university degree, having been born outside of Germany, belonging to the “higher risk (CAI)” or the “highest risk (CAI + STI)” category, having friends or acquaintances living with HIV, and attributing to PrEP a higher risk of infection with other STIs. In our multivariable analysis, belonging to the “higher risk (CAI)” or “highest risk (CAI + STI)” sexual risk behaviour category was a strong positive predictor of having a history of PrEP use. Further positive predictors were having a university degree, one or two parents born outside of Germany, and friends or acquaintances living with HIV, as well as attributing to PrEP a higher risk of infection with other STIs (Table 5). A response tree for the multivariable regression model for history of PrEP use is shown in Fig 2.

**Table 5:** ORs and 95% CIs for having a history of PrEP use, by education, family origins, sexual risk behaviour, having friends or acquaintances living with HIV, and risks attributed to PrEP use.

			Participants who had a history of PrEP use		Crude OR	Adjusted OR^†^
Participant characteristics		*N*^‡^	*n* (%)	*p* value^§^	(95% CI)	(95% CI)
**Education**				0.014		
	No university degree	154	16 (10.4%)		Reference	Reference
	University degree	305	61 (20.0%)		2.21 (1.21–-4.04)	2.44 (1.22–4.91)
**Family origins**				0.031		
	Participant and parents born in Germany	279	37 (13.3%)		Reference	Reference
	One or two parents born outside Germany	70	17 (24.3%)		1.92 (0.98–3.78)	3.03 (1.37–6.73)
	Participant born outside Germany	112	24 (21.4%)		1.82 (1.02–3.24)	1.80 (0.90–3.60)
**Sexual risk behaviour (past six months)**				<0.001		
	No STI; no multiple* CAI partners	276	18 (6.5%)		Reference	Reference
	STI; no multiple* CAI partners	30	0 (0.0%)		Empty	Empty
	No STI; multiple* CAI partners	103	31 (30.1%)		6.92 (3.57–13.43)	7.25 (3.64–14.45)
	STI; multiple* CAI partners	52	29 (55.8%)		19.10 (9.04–40.35)	16.18 (7.37–35.53)
**Having friends or acquaintances living with HIV**				<0.001		
	No	116	5 (4.3%)		Reference	Reference
	Yes	344	73 (21.2%)		5.66 (2.22–14.41)	4.16 (1.53–11.37)
**“A higher risk of getting infected with STIs”**				0.013		
	Not selected as risk seen for people using PrEP	165	17 (10.3%)		Reference	Reference
	Selected as risk seen for people using PrEP	302	64 (21.2%)		2.35 (1.28–4.30)	2.77 (1.39–5.52)

CAI, condomless anal intercourse; CI, confidence interval; OR, Odds ratio; PrEP, pre-exposure prophylaxis; STI, sexually transmitted infection. P-values from joint Wald tests of the null hypothesis that there is no variation across a category for the univariate regressions and the multivariate regression model were 0.0068 and 0.0120 for education, 0.0537 and 0.0170 for family origins, < .0001 and < .0001 for sexual risk behaviour, <0.0001 and 0.0054 for having friends or acquaintances living with HIV, and 0.0034 and 0.0039 for attributing PrEP a higher risk of getting infected with STIs, respectively.

^†^Multivariable analysis adjusting for education, family origins, sexual risk behaviour, having friends or acquaintances living with HIV, and risk of STI attributed to PrEP intake.

^‡^The sample excludes patients who were missing information on the relevant variables. Fig 2 gives an overview of participants included and excluded in the regression model.

^§^From Chi-squared tests of the null hypothesis that there is a no significant difference between the expected frequencies and the observed frequencies in one or more categories (e.g., across sexual risk behaviour groups).

*"multiple" was defined as reporting having had two or more CAI partners in the past six months.

### Anticipated impact of PrEP on participants’ use of condoms

When asked about the extent to which they agreed with the statement that they had (or would have) anal sex without a condom more often when taking PrEP, 45.4% of the participants agreed or strongly agreed whereas 33.0% disagreed or strongly disagreed. Participants who expressed a desire to use PrEP and those who stated that they were using or had used PrEP were significantly more likely to agree with the statement than other participants (Table 6).

**Table 6:** Anticipated impact of taking PrEP on participants’ use of condoms, by desire to use PrEP and history of PrEP use.

“I have (or would have) anal sex without a condom more often when taking PrEP”						
	*“I would like to use PrEP myself”*		p value^§^	*History of PrEP use*		p value^§^
	Agree or strongly agree (N = 207)	Neutral, disagree or strongly disagree (N = 211)		Yes (N = 80)	No (N = 372)	
Strongly disagree	18 (8.7%)	58 (27.5%)	< .001	7 (8.8%)	77 (20.7%)	0.002
Disagree	36 (17.4%)	32 (15.2%)		7 (8.8%)	64 (17.2%)	
Neither agree nor disagree	23 (11.1%)	38 (18.0%)		8 (10.0%)	55 (14.8%)	
Agree	79 (38.2%)	64 (30.3%)		37 (46.3%)	116 (31.2%)	
Strongly agree	39 (18.8%)	13 (6.2%)		17 (21.3%)	42 (11.3%)	
I never use condoms anyway	12 (5.8%)	6 (2.8%)		4 (5.0%)	18 (4.8%)	

^§^From Chi-squared tests of the null hypothesis that there is a no significant difference between the expected frequencies and the observed frequencies in the categories.

### Attitudes towards insurance coverage and pricing of PrEP

The majority of participants stated that the cost of PrEP should be covered by public health insurance in Germany, either for all MSM who want to use PrEP (64.7%) or only for MSM at the highest risk of acquiring HIV (13.4%). For the majority of participants (59.1%), an acceptable price per month if PrEP were never to be covered by public health insurance was 50 euros or less, followed by 100 euros or less for 21.3%, and 200 euros or less for 6.2%.

## Discussion

Our study is the first to use a facility-based survey to investigate what MSM in Berlin know about PrEP, the extent to which and how they use it, and the attitudes they have towards it. We additionally sought to identify factors associated with participants’ willingness to use PrEP and any history of PrEP use.

With data provided by almost 500 MSM in Berlin with a self-reported negative or unknown HIV serostatus, we found that awareness of PrEP, at 90%, was very high. However, fewer than half of those who were aware of PrEP felt well informed about it. This is troubling given that at least 60% of participants who were currently on PrEP or had used it at some point in the past reported that they had obtained some or all of their medication from an informal source, such as imports, pills originally prescribed for PEP or a friend’s HIV medication. The individual and public health risks of informal PrEP use are manifold and include delays in identifying side effects and infections with other STIs, an increased risk of HIV infection, and the development of drug resistant HIV strains that can be transmitted to others and harder to treat. To address this situation it will be crucial for policy makers in Germany to consider recent calls from the German STI Association (DSTIG) and the DAIG to improve access to PrEP and PrEP education through regular health care services [40,41]. As of July 2018, public health insurance in Germany did not cover the cost of PrEP medication or any related diagnostic tests or patient education specifically related to PrEP.

The importance of being able to obtain PrEP through formal channels is further supported by another of our findings, namely that participants who reported that they were taking PrEP in a manner consistent with the evidence (i.e., regularly or on-demand) were significantly more likely to have obtained a prescription from their doctor. Indeed, reliable information on PrEP would appear to be an important enabler of its proper use considering that participants who felt they were well informed about PrEP were better at identifying the true risks associated with it. More generally, the likelihood of having a history of PrEP use was associated in our regression model with having a university degree and with having friends or acquaintances who are living with HIV. Information may play a role as an enabler in both scenarios if we assume that MSM who have a university degree may have better access to health information or seek it out more assertively than those who do not. Likewise, it is possible that being part of a social network in which people communicate more openly about HIV leads to greater awareness of the disease and facilitates access to information about HIV medication and prevention.

Conversely, a lack of information was cited as a barrier to PrEP use by almost one third of participants who had no history of PrEP intake. Given that almost half of this group also indicated that they would consider using PrEP if they had fewer worries about side effects, it would seem that efforts to improve PrEP education in Germany should focus on the potential side effects of PrEP therapy in addition to emphasising the importance of adherence and regular diagnostic testing.

The most frequent sources of information about PrEP for our participants were their friends and acquaintances, as well as magazines, blogs and dating apps. This is unsurprising given evidence pointing to the important role of word-of-mouth communication when people make health-related decisions [42]. Given the high proportion of informal PrEP use suggested by our data, however, the fact that only a quarter of MSM in our sample reported doctors as one of their sources of information about PrEP is concerning. One explanation may be that doctors in Berlin are targeting information at the individuals they feel will benefit the most from PrEP. This is supported by our finding that those at the highest risk of HIV infection were significantly more likely to name their doctor as a source of information about PrEP than those at low risk.

Targeting information to a small group of MSM might also be a way for doctors in Berlin (and Germany as a whole) to cope with a system of payment for office-based health professionals that often makes it difficult to recover the cost of consultations as lengthy as those needed to educate patients about preventive measures such as PrEP [43,44]. However, one of the goals of PrEP provision is to avoid informal use of the medications. Our study provides some evidence that this targeting, if it is taking place, may be too narrow, leading to unintended negative consequences for individual and public health as detailed above. These concerns are further underscored by our finding that almost one quarter of participants who had no history of PrEP use reported that they had had condomless anal intercourse with more than one partner over the past six months.

The cost of PrEP is described frequently in the literature as a barrier to its use [45–47]. We therefore included a question in our survey to identify whether cost might be seen as a barrier by participants in our sample. However, because the price of a month’s supply of generic PrEP in Germany fell from approximately 600 euros to as low as 50 euros during the study period, our data on this question are of limited validity. Regardless, the majority of participants indicated that 50 euros per month was an acceptable price if PrEP continued not to be covered by public health insurance. Interestingly, most participants also felt that PrEP should be covered by public health insurance for all MSM who wanted to use it, regardless of their HIV risk. This suggests that participants may see access to PrEP as a matter of equality in contrast to what may be a narrowly targeted approach among doctors, as discussed above. In fact, almost one third of participants with no history of PrEP use indicated that they would consider using PrEP if a doctor prescribed it for them.

In addition to being more likely to receive information about PrEP from their doctors, participants at higher risk of HIV infection because of multiple CAI partners, or because of multiple CAI partners and an STI diagnosis, were much more likely to express the desire to take PrEP or to have a history of PrEP use than MSM at low risk. This is encouraging, both from an individual and public health perspective, yet it again raises the issue of informal PrEP use. Almost half of our participants stated that they had, or would have, anal sex without a condom more often when taking PrEP, thus increasing their risk of infection with other STIs. Moreover, those who expressed a desire to take PrEP or had a history of PrEP use were significantly more likely than those who did not to report that they did or would engage in CAI. While STIs can be detected and treated early among PrEP users who are well integrated into a regular STI testing scheme, this is rather unlikely for people who obtain PrEP through informal channels or take it irregularly.

We found evidence that MSM with family but not personal origins outside Germany were significantly more likely to have a history of PrEP use than MSM with family origins within Germany. Unfortunately, participants rarely specified which countries their non-German-born parent or parents came from. We therefore cannot draw any conclusions about whether this subgroup is representative of MSM whose families come from the historical source countries of migration to Berlin, such as Turkey, Poland and Russia [48]. Further studies are necessary to assess whether the needs of ethnic minorities in Berlin and Germany as a whole are being adequately met. The same applies to MSM in lower income groups, who are probably underrepresented in our sample. The state government of Berlin is planning to target this specific group with a model project that should provide free PrEP services to a limited number of financially deprived MSM [37].

Some of our findings are similar to those of an anonymous online survey of MSM conducted in Germany in 2016 [49]. The mean age of participants was the same, awareness of PrEP was similarly common, and similar proportions of participants reported having had an STI diagnosis within the past six months and being more likely not to use a condom when taking PrEP. Furthermore, the proportion of patients in our sample who reported obtaining PrEP only through a private prescription (32.1%) was similar to the proportion of patients in the sample of Spinner et al. [49] who reported accessing PrEP under medical supervision (29.2%). Moreover, the proportion of participants in both studies who reported obtaining at least some of their PrEP through informal channels was similarly high at 60% to 70%.

While sexual risk behaviour was also identified by Spinner et al. as a predictor of having a history of PrEP use, they defined risk contacts as CAI under the influence of recreational drugs, whereas we collected data on the frequency of anal intercourse overall and of CAI. Nevertheless, the similarities suggest that both study samples may be broadly representative of the broader population of MSM in Germany. This being said, the proportion of participants who reported a history of PrEP use in the survey by Spinner et al. [49] (7.5%) was considerably lower than in our sample. This in unsurprising, however, if we consider that PrEP uptake in a city like Berlin with a large population of MSM is likely to be higher than in Germany as a whole. In a survey of MSM in Amsterdam from 2015,[50] the proportion of participants aware of PrEP was much lower than that in our study. However, given the rapid developments in the field of PrEP, such as growing evidence to support its efficacy and safety, efforts to implement PrEP and reductions in price, this difference between data from 2015 and 2017/18 is similarly unsurprising.

This study has important limitations. First, when asking participants about their number of CAI partners, we did not distinguish between insertive and receptive CAI, although the risk of infection clearly differs between the two. However, we were interested primarily in obtaining data that could be grouped and analysed according to the CDC recommendations for PrEP use. Second, like other sampling strategies, facility-based sampling introduces a selection bias that can limit the external validity of findings [51–53]. While a strength of our sample is its broad age range (18–79 years), it likely reflects the part of the MSM community in Berlin that is well integrated within and seeking information from LGBTI counselling centres and HIV-specialist practices. This may help explain the high proportion of university degrees among our participants and the low proportion of participants who reported that they or their parents had been born in the countries with the historically highest flows of migration to Berlin. It is therefore important to consider that our sample may not include MSM in lower income groups or who are facing cultural barriers to access and might have the greatest need for information and, indeed, PrEP services. Moreover, it is likely that some of the participating doctors did not, as they had been asked, invite all eligible patients to take part in the survey. This may have led to patients being more likely to have been included if they asked about PrEP of their own volition and therefore to selection bias. A third important limitation is that, while we did not exclude transgender MSM from participating in the survey, we did not explicitly instruct participating centres to include this group, nor did we measure how many transgender MSM may have taken part. Other sampling strategies would have been necessary to obtain meaningful data on transgender MSM’s attitudes towards PrEP but would have gone beyond the scope of our study. Fourth, although we attempted to recruit several HIV specialist practices in former East Berlin, only one of these chose to participate. It was very centrally located and may not cater to many patients on the eastern outskirts of the city, where there are larger numbers of people with a family history of migration from the former Soviet Union and Vietnam [48]. Obtaining a representative sample of minorities, particularly sexual ones, remains a challenge. Nevertheless, many of the sociodemographic characteristics in our sample are comparable to those among participants in earlier, online surveys of MSM in Germany [49,54]. The mean age of our participants and the proportion of those who reported that they or their parents had been born outside of Germany were similar to the figures recorded by the participating sexual health counselling centres in 2016 as part of their routine data collection (mean age: 34.2 years; 52.2% born themselves or with parents born outside Germany) [55].

Lastly, we could not assess patterns of non-response because we had no information on the total number of patients or clients who were invited to participate in the survey versus the number who declined. However, as is the case for all epidemiological research, the size of the observed associations is important. In our study, the relatively high response rate for this type of research, the multivariable analysis used and the large size of the observed associations, particularly for sexual risk behaviour, suggest that our findings are not likely to result from non-response bias alone.

Our post-estimation regression diagnostics, including tests for multicollinearity and potentially influential observations, as well as sensitivity analyses including all variables of a priori interest, suggest that the findings of both regression models are robust.

## Conclusions

Our facility-based survey of almost 500 HIV-negative MSM in Berlin found a very high level of awareness of PrEP but also a strong need for more education on its pros, cons and proper use. From an individual and public health perspective, this need should be regarded as acute given that almost one quarter of our participants who reported never having used PrEP also reported having had condomless anal intercourse with more than one partner in the past six months. Moreover, at least 60% of participants who reported using PrEP had obtained some or all of it through informal channels, making it less likely that they were always taking their medication under medical supervision. We also found evidence that doctors in Berlin might be sensibly targeting the provision of PrEP services at those with the highest risk of HIV infection, but that this targeting could be too narrow, allowing some people to fall through the gaps. If the Berlin state government intends to go beyond its commitments as part of the Fast-Track Cities initiative, policy makers at the state and federal levels will need to consider recent calls from the German STI Association to improve access to PrEP and PrEP education through regular health services.

## Supporting information

S1 File English language questionnaire. (PDF)

S2 File German language questionnaire. (PDF)

S3 File Minimal underlying data set and codebook. Age of respondents and qualitative data have been removed to ensure patient anonymity.
(XLSX)
