# Supplementary material for: Knowledge and use of HIV pre-exposure prophylaxis among men who have sex with men in Berlin – A multicentre, cross-sectional survey
Source: PLoS One. 2018 Sep 13;13(9):e0204067. doi: 10.1371/journal.pone.0204067 (PMC6136827; doi:10.1371/journal.pone.0204067)
Supplement: S1 File — (PDF) [file pone.0204067.s001.pdf]

CHARITÉ

ChartéCentrum für Innere Medizin und Dermatologie

**Survey on what men who have sex with men know and think about HIV Pre-Exposure Prophylaxis (PrEP)**

We would like to give you the opportunity to participate in a survey on sexual health.

**The survey is anonymous and takes 10 minutes to complete. When you are done, please place the questionnaire in the box provided.**

Your participation is voluntary. You can decide not to participate at any point for whatever reason with no adverse consequences.

**Please only take part in this survey if you are a man aged 18+ years and if you have sex with men. Please complete the survey only once.**

**What is the survey about?**

For some time now it has been possible to take a pill to prevent getting infected with HIV. This is called HIV pre-exposure prophylaxis (PrEP). HIV-negative men who have sex with men can use it to protect themselves from an HIV infection even when not using a condom. The pill Truvada® has been licensed for this purpose in Germany since August 2016. In the meantime, generic versions of Truvada® have become available.

In Germany, people who use PrEP have to pay the cost themselves. Since December 2017 it has been available from 69.90 euros per month (daily intake). As part of a special programme, PrEP can also be purchased for 50 euros per month. In studies, other PrEP medications are being tested, such as long-acting injectables that are administered only every 1 to 3 months.

We are conducting this survey because we are interested in knowing how well informed men who have sex with men are about PrEP and under what circumstances they might consider taking it themselves. We would also like to know which factors influence what you know and think about PrEP.

**Data protection**

**We guarantee your anonymity and will not collect any information about you that would allow anyone to identify you personally.** The completed, anonymous questionnaires will be sent to the Department of Dermatology, Venereology and Allergy at Charité University Hospital in Berlin to undergo statistical analysis. A report of the survey will be published in 2018. No data will be published that would allow anyone to identify that you have taken part in the survey. The report will be published in a scientific journal. Further reports of the results may be presented at medical congresses, in magazines and on the websites of non-profit organizations or charities working on the topic of HIV and sexual health.

**Who are we?**

We are a group of doctors and researchers who work at the Department of Dermatology, Venereology and Allergy at Charité University Hospital and in different areas of health care in Berlin. We are interested in improving health care for men who have sex with men.

**If you have any questions please contact:**

Dr. med. R. N. Werner, Klinik für Dermatologie, Venerologie und Allergologie, Charité - Universitätsmedizin Berlin, Charitéplatz 1, 10117 Berlin, Tel.: +49 30 450 518313.

**Before taking part in this survey, did you know what PrEP is?**

- O Yes
- O No

**If you already knew what PrEP is, where did you get that knowledge from?**
*(multiple answers allowed)*

- O Not applicable (I didn't know about PrEP)
- O Friends / acquaintances
- O Doctor
- O Counselling center
- O Magazine, journal or blog
- O Dating app or platform
- O Elsewhere: ___________________________

*Do you agree or disagree with the following statement?* **"I am well informed about PrEP"**

- O Strongly disagree
- O Disagree
- O Neither agree nor disagree
- O Agree
- O Strongly agree

**Have you ever used PrEP?**

- O No
- O Yes, but not on a regular basis
- O Yes, I regularly use it before and after risky sex (as needed)
- O Yes, I use it continuously (daily pill)

**If you have ever used PrEP, where did you get it from?** *(multiple answers allowed)*

- O Not applicable (I have never used it)
- O A doctor prescribed it (private prescription)
- O Imported from another country (for example from the UK as a generic pill)
- O From pills I received for post-exposure prophylaxis (PEP)
- O From a friend's HIV medication
- O Other way: ___________________________

*Do you agree or disagree with the following statement?*
**"I would like to use PrEP myself"**

- O Not applicable (I already take PrEP)
- O Strongly disagree
- O Disagree
- O Neither agree nor disagree
- O Agree
- O Strongly agree

**Under what circumstances would you use PrEP?** *(multiple answers allowed)*

- O Not applicable (I already take PrEP)
- O I wouldn't use it under any circumstances
- O If I had more information
- O If I had fewer worries about side effects
- O If a doctor prescribed it
- O If it was cheaper
- O Other circumstance: ___________________

**If you are considering or already using PrEP, how would you prefer to take it?**

- O Not applicable (I am not considering it)
- O Continuously (daily pill)
- O Before and after risky sex (as needed)
- O As an injection every few months
- O Not sure

*Do you agree or disagree with the following statement?* **"Overall, PrEP is a safe way to prevent an infection with HIV"**

- O Strongly disagree
- O Disagree
- O Neither agree nor disagree
- O Agree
- O Strongly agree

**What risks do you see for people who use PrEP?** *(multiple answers allowed)*

- O None
- O Mild / temporary side effects
- O Severe / permanent side effects
- O A higher risk of getting infected with HIV
- O A higher risk of getting infected with other sexually transmitted infections
- O Other risks: ___________________________
- O Not sure

*Do you agree or disagree with the following statement?* **"I have (or would have) anal sex without a condom more often when taking PrEP"**

- O Not applicable (I never use condoms anyway)
- O Strongly disagree
- O Disagree
- O Neither agree nor disagree
- O Agree
- O Strongly agree

**If you are considering or already using PrEP, what is your main reason for this?** *Please specify:*

______________________________________________________________________

*Do you agree with the following statement?*
**"I think PrEP should be paid for by German public health insurance"**

- O No
- O Yes, but only for men who are at high risk of getting infected with HIV
- O Yes, for all men who have sex with men and want to use PrEP
- O Not sure

**If German public health insurance continues NOT to pay for PrEP, what price per month do you think is acceptable?**

- O Up to 50 euros
- O Up to 100 euros
- O Up to 200 euros
- O Up to 300 euros
- O Up to 400 euros
- O Up to 500 euros
- O Up to 600 euros
- O Up to 700 euros
- O Up to 800 euros
- O Not sure

**When did you last get tested for HIV?**

- O Approximately _____/_____ (month / year)
- O I've never been tested
- O I don't know

**What do you think your current HIV status is?**

- O I don't have HIV
- O I am HIV positive
- O I don't know

**Have you been diagnosed with a sexually transmitted infection in the last 6 months?**

- O No / not that I know of
- O Yes

**How would you describe your role when you have anal sex with another man?**

- O I don't engage in anal sex
- O I am bottom only
- O I am more bottom than top
- O I am top and bottom (versatile)
- O I am more top than bottom
- O I am top only

*Do you agree or disagree with the following statement?* **"When I have sex, it is always as safe as I'd like it to be"**

- O Strongly disagree
- O Disagree
- O Neither agree nor disagree
- O Agree
- O Strongly agree

**Do you have HIV-positive friends or acquaintances?**

- O Yes, close friends
- O No
- O Yes, acquaintances

**How many men have you had anal sex with in the last 6 months?**

- O None
- O 1
- O 2 to 5
- O 6 to 10
- O More than 10

**How many men have you had anal sex with in the last 6 months <u>without using a condom</u>?**

- O None
- O 1
- O 2 to 5
- O 6 to 10
- O More than 10

**How old are you?** _______ years

**Where do you currently live?**

- O Berlin
- O Other city in Germany: ________________
- O Small town / countryside in Germany: ____
- O Other country: ____________(city/country)

**What is the highest degree or level of school you have completed?**

- O Primary education
- O Secondary education lasting up to year 10 (or similar)
- O Secondary education with apprenticeship
- O Secondary education lasting up to year 12 (for example A Levels, high school diploma)
- O University degree (for example: Bachelor's or Master's degree)

**How do you feel about your finances?**

- O I don't always have enough money to pay for the things I need
- O I have enough money to pay for the things I need
- O I have more than enough money to pay for the things I need

**What are your origins?**

- O My parents <u>and</u> I were born in Germany
- O <u>One</u> of my parents was born outside of Germany, in: ________________________
- O <u>Both</u> of my parents were born outside of Germany, in: ________________________
- O <u>I</u> was born outside of Germany, in: ____________________________________

**Many thanks for your time and participation in the survey!**
