# Supplementary material for: Knowledge and use of HIV pre-exposure prophylaxis among men who have sex with men in Berlin – A multicentre, cross-sectional survey
Source: PLoS One. 2018 Sep 13;13(9):e0204067. doi: 10.1371/journal.pone.0204067 (PMC6136827; doi:10.1371/journal.pone.0204067)
Supplement: S2 File — (PDF) [file pone.0204067.s002.pdf]

CHARITÉ

ChariteCentrum für Innere Medizin und Dermatologie

Umfrage: Kenntnisse und Einstellungen zur HIV-Präexpositionsprophylaxe (PrEP) unter Männern, die Sex mit Männern haben

Wir möchten Dir die Teilnahme an einer Studie zum Thema sexuelle Gesundheit anbieten.

Die Studie besteht aus einem anonymen Fragebogen – es dauert etwa 10 Minuten, den Fragebogen auszufüllen. Wenn Du fertig bist, wirf den Fragebogen in die bereitgestellte Box ein.

Die Teilnahme ist freiwillig. Du kannst Deine Teilnahme jederzeit ohne Angabe von Gründen beenden. Wenn Du nicht teilnehmen möchtest oder Deine Teilnahme zurückziehst, hast Du keine Nachteile zu befürchten.

Bitte nimm an dieser Umfrage nur teil, wenn Du volljährig und männlich bist, und Sex mit Männern hast. Bitte fülle diesen Fragebogen nur einmal aus.

Worum geht es?

Seit einiger Zeit gibt es die Möglichkeit, präventiv (also vorsorglich) Medikamente einzunehmen, um zu verhindern, dass man sich mit HIV infiziert. Dies wird als HIV-Präexpositionsprophylaxe (PrEP) bezeichnet. So können HIV-negative Männer, die Sex mit Männern haben, sich auch bei Sex ohne Kondom vor einer Infektion mit HIV schützen. Das Medikament Truvada® ist seit August 2016 in Deutschland für diesen Zweck zugelassen. Mittlerweile gibt es auch Generika von Truvada®.

Die PrEP muss von den Nutzern selbst bezahlt werden und ist seit Dezember 2017 zu einem Preis ab 69,90 Euro pro Monat (bei täglicher Einnahme) verfügbar. Im Rahmen eines Sondermodells kann die PrEP auch zu einem Preis von 50 Euro pro Monat bezogen werden. Weitere PrEP-Medikamente werden zur Zeit in Studien erprobt, darunter auch langwirksame Spritzen, die nur alle 1-3 Monate verabreicht werden müssen.

Uns interessiert, wie gut sich Männer, die Sex mit Männern haben, mit der PrEP auskennen, und ob und unter welchen Voraussetzungen die PrEP für sie selbst in Frage kommt. Hierbei möchten wir auch gerne wissen, welche Faktoren die Kenntnisse und Einstellung zur PrEP beeinflussen.

Datenschutz

Wir garantieren Deine Anonymität und werden keine Informationen über Dich erheben, durch die Du persönlich identifiziert werden kannst. Die ausgefüllten anonymen Fragebögen werden an die Klinik für Dermatologie, Venerologie und Allergologie an der Charité – Universitätsmedizin Berlin übermittelt und dort statistisch ausgewertet. Ein Bericht der Umfrage wird Mitte 2018 erstellt. Hier werden keine Daten veröffentlicht, die einen Rückschluss auf die einzelnen Teilnehmer ermöglichen. Der Bericht wird in einer wissenschaftlichen Zeitschrift publiziert werden. Möglicherweise werden weitere Berichte von den Umfrageergebnissen auf medizinischen Kongressen, in Magazinen und/oder auf den Internetseiten der AIDS-Hilfe oder anderer gemeinnütziger Einrichtungen veröffentlicht.

Wer sind wir?

Wir sind eine Gruppe von Medizinern und Gesundheitswissenschaftlern, die an der Klinik für Dermatologie, Venerologie und Allergologie der Berliner Charité – Universitätsmedizin und in verschiedenen Bereichen der Gesundheitsversorgung tätig sind. Uns interessiert die Frage, wie die Gesundheitsversorgung von Männern, die Sex mit Männern haben, verbessert werden kann.

Kontakt für Rückfragen

Dr. med. R. N. Werner, Klinik für Dermatologie, Venerologie und Allergologie, Charité - Universitätsmedizin Berlin, Chariteplatz 1, 10117 Berlin, Tel.: +49 30 450 518313.

Bevor Du an dieser Umfrage teilgenommen hast, wusstest Du was PrEP ist?

O Ja
O Nein

Wenn Du PrEP schon kanntest oder schon mal davon gehört hast, woher?
(Mehrfachnennung möglich)

O Nicht zutreffend (kannte ich nicht)
O Freunde / Bekannte
O Arzt / Ärztin
O Beratungsstelle
O Magazin, Zeitschrift oder Blog
O Dating-App oder -Portal
O Anderswo: ___________________________

Wie sehr stimmst Du der Aussage zu?
„Ich kenne mich gut mit PrEP aus."

O Stimme gar nicht zu
O Stimme eher nicht zu
O Weder noch
O Stimme eher zu
O Stimme voll zu

Hast Du selbst schon mal PrEP genommen?

O Nein
O Ja, aber nicht regelmäßig
O Ja, regelmäßig anlassbezogen vor und nach risikoreichem Sex (bei Bedarf)
O Ja, ich nehme PrEP kontinuierlich (täglich) ein

Wenn Du selbst schon mal PrEP eingenommen hast, wie hast du sie bekommen?
(Mehrfachnennung möglich)

O Nicht zutreffend (noch nie eingenommen)
O Von einem Arzt auf Privatrezept
O Aus einem anderen Land importiert (z.B. als Generikum aus Großbritannien)
O Im Rahmen einer Postexpositionsprophylaxe (PEP)
O Ich habe Medikamente von einem HIV-positiven Freund / Bekannten genommen
O Anders: _____________________________

Wie sehr stimmst Du der Aussage zu?
„Ich würde gerne PrEP einnehmen"

O Nicht zutreffend (nehme ich schon ein)
O Stimme gar nicht zu
O Stimme eher nicht zu
O Weder noch
O Stimme eher zu
O Stimme voll zu

Unter welchen Voraussetzungen würdest Du die PrEP einnehmen?
(Mehrfachnennung möglich)

O Nicht zutreffend (nehme ich schon ein)
O Unter keinen Voraussetzungen
O Wenn ich mehr Informationen hätte
O Wenn ich weniger Sorgen vor Nebenwirkungen hätte
O Wenn sie mir ein Arzt verschreiben würde
O Wenn die PrEP billiger wäre
O Andere Voraussetzung: _________________

Wenn Du überlegst, PrEP zu nehmen oder PrEP schon nimmst, welche Art der Einnahme würdest Du bevorzugen?

O Nicht zutreffend (möchte ich nicht nehmen)
O Tägliche Einnahme einer Tablette
O Anlassbezogen vor und nach risikoreichem Sex (bei Bedarf)
O Langwirksame Spritze (z.B. alle zwei Monate)
O Weiß ich nicht

Wie sehr stimmst Du der Aussage zu?
„Insgesamt ist PrEP eine sichere Option, sich vor HIV zu schützen."

O Stimme gar nicht zu
O Stimme eher nicht zu
O Weder noch
O Stimme eher zu
O Stimme voll zu

Welche Risiken siehst Du für Menschen, die PrEP einnehmen?
(Mehrfachnennungen möglich)

O Keine
O Leichte / vorübergehende Nebenwirkungen
O Schwere / bleibende Nebenwirkungen
O Höheres Risiko, sich mit HIV zu infizieren
O Höheres Risiko, sich mit anderen sexuell übertragbaren Erkrankungen zu infizieren
O Andere: _____________________________
O Weiß ich nicht

Wie sehr stimmst Du der Aussage zu?
„Ich hätte bzw. habe mit PrEP öfter Analverkehr (Ficken) ohne Kondom."

O trifft nicht zu (ich nutze sowieso nie Kondome)
O Stimme gar nicht zu
O Stimme eher nicht zu
O Weder noch
O Stimme eher zu
O Stimme voll zu

Wenn Du überlegst, PrEP zu nehmen oder sie schon nimmst, was ist Deine Hauptmotivation dafür? *Bitte eintragen:*

____

Stimmst Du der Aussage zu?
„Ich finde, die PrEP sollte von der gesetzlichen Krankenkasse bezahlt werden.“
O Nein
O Ja, aber nur für Männer, die ein hohes Risiko haben, sich mit HIV zu infizieren
O Ja, für alle Männer, die Sex mit Männern haben, und die PrEP nehmen wollen
O Weiß ich nicht

Wenn die PrEP weiterhin NICHT von den Krankenkassen bezahlt wird, welchen Preis pro Monat findest Du akzeptabel?
O Bis 50 Euro O Bis 500 Euro
O Bis 100 Euro O Bis 600 Euro
O Bis 200 Euro O Bis 700 Euro
O Bis 300 Euro O Bis 800 Euro
O Bis 400 Euro O Weiß ich nicht

Wann war Dein letzter HIV-Test?
O Circa _________/_________ (Monat / Jahr)
O Ich habe mich noch nie testen lassen
O Weiß ich nicht mehr

Was denkst Du, ist Dein derzeitiger HIV-Status?
O Ich bin HIV-negativ
O Ich bin HIV-positiv
O Weiß ich nicht

Hattest Du in den letzten 6 Monaten eine sexuell übertragbare Erkrankung?
O Nein / Nicht, dass ich wüsste
O Ja

Wenn Du mit Männern Analverkehr hast (Ficken), wie würdest Du Dein Verhalten beschreiben?
O Ich habe keinen Analverkehr
O Ich bin nur passiv
O Ich bin eher passiv
O Ich bin aktiv und passiv gleichermaßen
O Ich bin eher aktiv
O Ich bin nur aktiv

Wie sehr stimmst Du der Aussage zu?
„Wenn ich Sex habe, ist das immer so safe, wie ich es gerne hätte.“
O Stimme gar nicht zu
O Stimme eher nicht zu
O Weder noch
O Stimme eher zu
O Stimme voll zu

Hast Du HIV-positive Freunde / Bekannte?
O Ja, Freunde O Nein
O Ja, Bekannte

Mit wie vielen unterschiedlichen Männern hattest Du in den letzten 6 Monaten Analverkehr?
O Mit keinem O Mit 6 bis 10
O Mit einem O Mit mehr als 10
O Mit 2 bis 5

Mit wie vielen unterschiedlichen Männern hattest Du in den letzten 6 Monaten Analverkehr <u>ohne Kondom</u>?
O Mit keinem O Mit 6 bis 10
O Mit einem O Mit mehr als 10
O Mit 2 bis 5

Wie alt bist Du? _______ Jahre

Wo wohnst Du?
O Berlin
O Andere Stadt in Deutschland: ___________
O Ländliche Region in Deutschland: ________
O Anderes Land: ________________________(Stadt / Land)

Was ist Dein höchster Schul- oder Bildungsabschluss?
O Kein Schulabschluss
O Haupt-/Real-/Volksschulabschluss
O Lehrabschluss / zweijährige Ausbildung
O Abitur (Hoch- oder Fachhochschulreife)
O Universitäts- / Fachhochschulabschluss

Wie schätzt Du Deine finanziellen Verhältnisse ein?
O Ich habe Probleme über die Runden zu kommen
O Ich komme zurecht
O Ich komme gut zurecht

Hast Du einen Migrationshintergrund?
O Nein, ich <u>und</u> meine Eltern sind in Deutschland geboren
O Ja, <u>ein</u> Elternteil ist nicht in Deutschland geboren, sondern in __________________
O Ja, <u>beide</u> Eltern sind nicht in Deutschland geboren, sondern in __________________
O Ja, <u>ich</u> bin selbst nicht in Deutschland geboren, sondern in __________________

Vielen Dank für Deine Zeit und Mithilfe bei diesem Projekt!
